# Copy Number Analysis of Complement *C4A*, *C4B* and *C4A* Silencing Mutation by Real-Time Quantitative Polymerase Chain Reaction

**DOI:** 10.1371/journal.pone.0038813

**Published:** 2012-06-21

**Authors:** Riitta Paakkanen, Hanna Vauhkonen, Katja T. Eronen, Asko Järvinen, Mikko Seppänen, Marja-Liisa Lokki

**Affiliations:** 1 Transplantation Laboratory, Haartman Institute, University of Helsinki, Helsinki, Finland; 2 Division of Cardiology, Department of Medicine, Helsinki University Central Hospital (HUCH), Hospital District of Helsinki and Uusimaa, Helsinki, Finland; 3 Division of Infectious Diseases, Department of Medicine, Helsinki University Central Hospital, Hospital District of Helsinki and Uusimaa, Helsinki, Finland; Louisiana State University, United States of America

## Abstract

Low protein levels and copy number variation (CNV) of the fourth component of human complement (C4A and C4B) have been associated with various diseases. High-throughput methods for analysing *C4* CNV are available, but they commonly do not detect the most common *C4A* mutation, a silencing CT insertion (*CTins*) leading to low protein levels. We developed a SYBR® Green labelled real-time quantitative polymerase chain reaction (qPCR) with a novel concentration range approach to address *C4* CNV and deficiencies due to *CTins*. This method was validated in three sample sets and applied to over 1600 patient samples. *CTins* caused *C4A* deficiency in more than 70% (76/105) of the carriers. Twenty per cent (76/381) of patients with a *C4A* deficiency would have been erroneously recorded as having none, if the *CTins* had not been assessed. *C4A* deficiency was more common in patients than a healthy reference population, (OR = 1.60, 95%CI = 1.02–2.52, *p* = 0.039). The number of functional *C4* genes can be straightforwardly analyzed by real-time qPCR, also with SYBR® Green labelling. Determination of *CTins* increases the frequency of *C4A* deficiency and thus helps to elucidate the genotypic versus phenotypic disease associations.

## Introduction

Deficiencies of complement component C4 isotypes, *C4A* (MIM+120810) and *C4B* (MIM *120820), have been associated with various autoimmune, inflammatory or infectious diseases as well as with mental disorders and cancer survival [1]–[4]. Phenotypic C4 deficiencies are caused by increased protein consumption or genetic deficiencies, which may, in turn, result from deletions, conversions or silencing mutations [5], [6]. Low copy number variation (CNV, less than 2 copies) causes part, but not all phenotypic C4 deficiencies as it detects deletions and conversions, but not the silencing mutations [7]. The most common mutation leading to *C4A* silencing is a two-nucleotide CT-insertion (*CTins* MIM+120810) in exon 29, codon 1213 and is virtually absent in *C4B* [8], [9].

Real-time quantitative polymerase chain reaction (qPCR) measures the amount of PCR products. It has been named as the “method of choice” for CNV analyses in spite of the limitations [10]–[12]. The current real-time qPCR methods for *C4* CNV determination use the TaqMan® probing [13]–[15]. The presence of *CTins* has not been assessed with qPCR before [16]–[19].

In this study we describe and validate a sensitive and specific, low-cost real-time qPCR assay with SYBR® Green labelling for absolute quantification of CNV of *C4A*, *C4B* and *CTins* analyses. It has been successfully applied in parallel with C4 immunophenotyping to more than 1600 patient samples.

## Materials and Methods

### Samples

#### Internal validation

**Figure 1:**
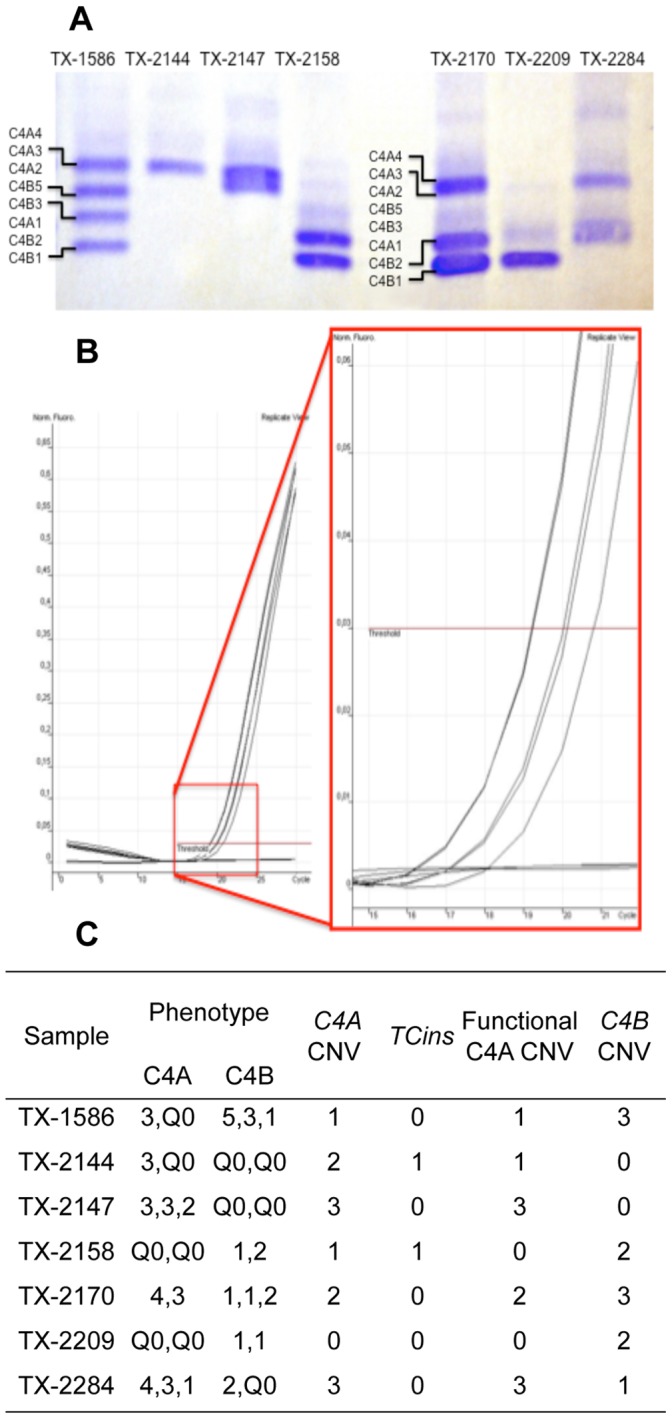
Method validation with selected samples. **A.** Immunophenotyping. The gel is skewed in the middle, leading to lower position of protein band levels on the left. All samples were analysed in a replicate run. **B.** Real-time quantitative PCR (qPCR) for copy number variation. The y-axis depicts the linear view of the fluorescence rate (from 0 to 0.6 in the full picture, from 0 to 0.06 in the magnification) and the x-axis the number of cycles (from 0 to 30 in the full picture and 15 to 21 in the magnification). Each curve represents the mean of two replicates of a sample. The lowest horizontal line represents non-template controls (sterile water), negative control and samples with zero copies of *C4B* having zero fluorescence due to undetectable amounts of DNA (TX-2144 and TX-2147). The curves from left to right depict samples with *C4B* CNV 3, 2 and 1 (TX-1586, TX-2170 and TX-2158, TX-2209 and TX-2284, respectively). The number of cycles at which the fluorescence curve cuts the threshold (the red horizontal line) is recorded; the greater amount of genes indicates the lower number of cycles surpassing the threshold. *C4A* and *CTins* qPCR runs resulted in similar output. **C.** Parallel results of C4 analyses. Functional *C4A* CNV was assessed by reducing the amount of *CTins* from *C4A* CNV.

Seven cases were collected from samples sent to our Laboratory. They were selected to cover the most common *C4A* and *C4B* CNVs, the presence of *CTins* and different genetic backgrounds of *C4A* deficiency (low CNV [samples 1586 and 2209] or silencing by *CTins* [samples 2144 and 2158], **Figure 1A and B**, **Supplementary Table S1**).

#### External validation

Samples with published *C4* CNVs were analysed to see whether qPCR results are applicable for samples processed by different DNA isolation methods. This was done in two different sample cohorts. Genomic DNA from 48 cell lines of consanguineous subjects with previously published *C4* CNV by real-time qPCR with TaqMan® dye [14] were purchased from the International Histocompatibility Working Group (IHWG) Cell Bank (Seattle, WA). Two samples (IHW09038 and 09102) were not available. Six samples with the most common *C4A* and *C4B* CNVs were used for method validation (**Supplementary Table S2**). Eighty-nine HapMap samples had been previously genotyped in parallel with three different methods, our SYBR® Green real-time qPCR, southern blotting and paralog ratio test [20].

#### Diagnostic routine

Between November 2004 and December 2009, 1648 samples of serum (2 ml) and peripheral EDTA blood (7 ml) were received in our accredited diagnostic HLA Laboratory for parallel qPCR CNV and immunophenotype analysis of C4A and C4B. These samples were considered to represent mainly infection prone (based on clinicians’ suspicion) patients as 60% of the samples were sent from the Division of Infectious Diseases in Helsinki University Central Hospital using such indications (personal communication, A. Järvinen).

The Ethics Committee, Department of Medicine**,** Hospital District of Helsinki and Uusimaa, waived the need for committee’s approval and patient consent because the used patient material was retrospectively and anonymously analysed as frequency data from our Diagnostic Laboratory’s result without patient identification, any interventions or contacts due to the current study. The results were commanded on clinical grounds from different institutions and used to guide patient care as seen suitable by the treating clinician.

### Sample Preparation

DNA was extracted from the buffy coat fraction of whole blood by NucleoSpin QuickPure columns (Machery-Nagel, Duren, Germany), diluted to 50 ng/µl in sterile water, and, after assessing purity (A260/A280>1.7), adjusted to 10 ng/µl (between 8.0 and 14.0 ng/µl) by spectrophotometer (NanoDrop® ND-1000, NanoDrop Technologies, Wilmington, DE, precision 0.1 ng/µl) for further use. Once diluted, the samples had to be stored at +8 C and analysed within 2–3 weeks. The purchased samples were extracted as described elsewhere, otherwise handled as above (http://locus.umdnj.edu/nigms/) [14].

### Primers and Master Mixes

The primers (Sigma Genosys, Heverhill, UK) were selected based on published sequences, allowing distinction between *C4A* and *C4B* on four of their five-base pair differences on exon 26 and detection of *CTins* in exon 29 (**Table 1**) [8], [9], [21], [22]. The primer and amplicon lengths, end specificity, G/C-contents, absence of secondary structures and Tm differences were optimized (**Table 1**).

**Table 1:** Primer sequences of *C4A*, *C4B, CTins* and *Beta-actin* qPCR runs.

qPCR	Primer	Specificity	Sequence 5′–3′^a^	SYBR® Green Mix	Annealing temperature (°C)	Amplicon size (bp)	Analysis threshold	Reference
C4A	C4A_F	*C4A*	AGG ACC CCT GTC CAG TGT TAG AC	ABsolute™	55	247	0.03	[22]
	C4A/B_R	*C4*	CAC TCT CTG CTT CAA TGG CT					[22]
C4B	C4B_F	*C4B*	AGG ACC TCT CTC CAG TGA TAC A	Brilliant®	57	247	0.03	[22]
	C4A/B_R	*C4*	CAC TCT CTG CTT CAA TGG CT					[22]
CTins	C4INS_ F	*CTins*	CTC TTC TCC CTG CCT TCC T	Brilliant®	57	88	0.1	[21]
	C4INS_ R	*CTins*	GCT CTG AGA ACC AGT GAC TGA GAG					[8], [9], [21]
Beta-actin	ACTIN_F	*Beta-actin*	GCA CTC TTC CAG CCT TCC	ABsolute™	60	435	0.05	
	ACTIN_R	*Beta-actin*	GCG CTC AGG AGG AGC AAT					

Abbreviations:

qPCR (real-time quantitative polymerase chain reaction),

*CTins* (CT-insertion mutation of *C4A* leading to non-expression).

a Specific bases are underlined.

Two SYBR® Green Master Mixes (ABsolute™ qPCR SYBR® Green Mix, AB-1159, ABgene, Epsom, UK and Brilliant SYBR® Green QPRC Master Mix, Staratagene, AH Diagnostics, Skärholmen, Sweden) with modified *Taq* polymerase having hot start capability were used depending on the analysis (**Table 1**).

### PCR Program

Real-time qPCR was performed with Rotor-Gene 3000 (Qiagen, Vienna, Austria). Reactions were adjusted to a final volume of 10 µl/well using 2 µl of genomic DNA (10 ng/µl), 0.25 µl of both of primers (20 pmol/µl), 2.5 µl of sterile water and 5 µl of Master Mix.

The qPCR program was as follows: Hot start at +95°C for 15 minutes, followed by 30 three-step cycles (15 seconds at +95°C, 45 seconds at annealing temperature and 45 seconds at +72°C). The annealing temperatures are indicated in **Table 1**.

Samples with known *C4* CNV (from 0 to 3 in *C4A* and *C4B* runs and from 0 to 1 in *CTins* run, patient samples with consistent immunophenotyping and qPCR results) served as controls. It is extremely rare to have two copies of *CTins* and therefore a control with two copies was used in a replication run when needed.

### Specificity Assessment

Other sequences were not specifically found, when performing the BLAST-search (www.ncbi.nih.gov/BLAST). Using the Primer-BLAST option, the mismatched primers did not produce similar sized amplicons.

A melt analysis (ramping from +65°C to +95°C, rising the temperature by 0.2°C at every step with 2-seconds interval), performed after the CNV runs, exhibited only one peak characterizing a homologous amplicon in *C4A* and *C4B* runs. For *CTins*, two individual non-overlapping peaks were detected, but the samples with the nonspecific peak at lower temperature did not surpass the threshold Ct in the actual qPCR run (data not shown).

### Data Analysis

Raw data were analysed using Rotor-Gene software v 6.0 (Qiagen, Vienna, Austria). Prior to the *C4* CNV analyses, the DNA concentration comparability between samples and controls was assured by the amplification of a housekeeping gene (beta-actin) in parallel with standard dilutions of 8, 10 and 14 ng/µl. Samples outside this range were discarded. The sample’s concentration was categorized as comparable (9–11 ng/µl, assuming control concentration 10 ng/µl), lower or higher than the control’s concentration. The use of concentration range was used to prevent the false interpretation of CNVs **(Supplementary Table S3**).

The run validity can be estimated by R^2^-value and reaction efficiency. The R^2^-value is used to depict how well the standard curve can be drawn from the given data. Reaction efficiency (percentage) can be assessed from standard curve.

Run validity was ensured by controls and adequate standard curves (R^2^>0.8). Outlier Ct-values resulting from nonspecific amplification were excluded.

The primary fluorescence data was normalized according to the Manufacturer’s instructions. Briefly, the first ten cycles were ignored and the background fluorescence was adjusted using Dynamic Tube Normalization-option. The threshold level of fluorescence was set to separate different CNVs by one Ct (**Table 1**).

The determination of CNV was performed by visual inspection, superimposing the sample’s trace on the controls’ traces. Samples with comparable concentration with the controls were recorded having the CNV of the closest trace (Ct difference <0.4). Samples with lower or higher concentration than the controls were rounded up or down, respectively. Samples with lower concentration and Ct values between 2 and 3 were rounded up to CNV 3. With higher concentrations, the values were rounded down to CNV 2. The same logic applies to other CNVs. High CNV numbers (4 or 5) with higher concentration than the controls were re-diluted (**Supplementary Table S3**). Samples with unclear CNVs were either re-assessed in beta-actin run with the controls or re-diluted from the stock.

The analysis program calculates CNVs by inserting the Ct-value into the standard curve equation and is used as a second opinion. Briefly, a linear standard curve equation is formed from controls’ Ct-values (cycle threshold, the number of cycles at which the sample’s trace exceeds an arbitrary threshold, values >26 considered as outliers) and logarithmic transformation of given concentration (with beta-actin analyse) or CNV (with C4 analyses). The unknown sample’s DNA quantity is calculated from the equation of standard curve with the obtained Ct value. *CTins* was reduced from the total *C4A* CNV.

### C4 Immunophenotyping

C4 immunophenotyping of diagnostic samples was carried out from serum as described (**Figure 1**) [23], [24]. One specialist (M-L.L.) analyzed all data independently of the genotyping results. The number of *C4* genes was estimated from protein bands and subsequently compared with the qPCR results in order to differentiate between the primary and secondary complement C4 deficiencies in a semi-quantitative fashion.

### Statistical Analysis

Statistical analyses were performed using PASW Statistics, version 18.0. Chi-square test with two-sided exact p-value was used at significance level <0.05. Cohen’s Kappa was calculated for C4 CNV values in methodologically satisfactory results in both geno- and phenotypic analyses (n = 1500 for C4A and n = 1542 for C4B). *C4* CNV frequencies were compared with reference population including samples with methodologically satisfactory result in all qPCR analyses (n = 1618).

## Results

### Internal Validation Shows No Overlap in Samples with Different Copy Numbers

The internal validation was performed with samples selected amongst diagnostic patients as the real-time qPCR optimization was performed with similarly processed samples (**Figure 1**). The qPCR analyses were replicated from two different dilutions, in five independent runs.

CNVs of a gene were consistently segregated by approximately one cycle in all replications. The inter-run and -dilution variance was very small (**Table S1**). Grouping all samples with a given CNV did not reveal any overlap in 95% confidence interval of Ct-values, even though different dilutions were combined (**Table 2**). The variance seemed to grow with increasing copy numbers, possibly due to greater sensitivity in sample manipulation in elevated concentrations. However, only *C4B* analysis showed increased variance in high CNVs, when samples were individually assessed (**Table S1**).

**Table 2:** Results of samples in method validation, detailed by copy numbers.

Copy number^a^ (n)		Replications (n)	Mean Ct value	95%CI	SD	Range	Variance (%)
C4A	0	12^b^					
	1	52	21.36	21.3–21.41	0.19	0.79	4
	2	52	20.4	20.33–20.46	0.24	1.21	6
	3	52	19.58	19.47–19.69	0.4	1.42	16
C4B	0	26					
	1	26	21.06	20.95–21.17	0.28	1.2	8
	2	52	20.25	20.17–20.33	0.28	1.14	8
	3	52	19.63	19.52–19.73	0.39	1.64	15
CTins	0						
	1	72	24.46	24.36–24.56	0.41	2.39	17

Abbreviations:

Ct (cycle threshold value, the number of cycles needed to surpass a threshold value, that is inversely related to the copy number of genes),

CI (confidence interval),

SD (standard deviation),

C4A, C4B (complement components C4A and B),

CTins (CT-insertion mutation of C4A leading to non-expression).

a For detailed results on individual samples, concentrations and runs, see Table S1.

b Fourteen Ct values >26 were discarded as outliers.

For run validity analyses, samples were assessed as controls with known CNVs for R^2^ and reaction efficiency determinations. The mean R^2^ values and reaction efficiencies were 0.88, 87.7% and 0.90, 115% for *C4A* and *C4B* runs, respectively (**Table S1**). For *CTins* runs these values were essentially similar (data not shown). Intercept values did not significantly differ between different runs (data not shown).

### Real-time qPCR is Applicable also to External Samples, but Controls have to be Isolated Using Similar Protocols to Ensure Reliable Results

In housekeeping gene analysis, the external samples were not comparable with our control samples, making the absolute quantification demanding (data not shown).

The six IHWG samples for validation were replicated from two dilutions, in eight separate analyses. The samples with different CNVs were seen to differ by one cycle with no overlap between different CNVs and total consistency with published *C4* CNV (**Table S2**). The Ct-values of a given CNV were close, but not comparable with the diagnostic samples’ values.

The *C4* CNV of the remaining 41/42 samples were concordant with previous results (98%, data not shown) [14]. The sample IHW09023 was reported to have zero copies of *C4A.* In our analyses, however, the CNV was repeatedly two. None of the studied samples carried *CTins*.

Of the 89 HapMap samples, 8 having consistent results in paralog ratio test and Southern blotting had discrepant results in real-time qPCR assay (data not shown) [20]. The samples might be degraded as the quality (A260/A280<1.7 *n* = 7) or concentration (beta-actin below the control dilutions *n* = 1) was inadequate, and as the discrepancies were all due to lower detected CNV by PCR. Retyping these samples by adjusting the concentration by approximation resulted in concordant results in 7/8 samples.

### Real-time qPCR and Immunophenotyping Exhibit Great Compatibility in Patient Samples

*C4A and C4B* CNV, *CTins* and serum C4A and C4B protein phenotypes were independently analyzed from 1648 patients.

For C4A, the results were unambiguous in 98.4% (1621/1648) for qPCR and in 92.4% (1523/1648) for immunophenotyping of the samples. For C4B, the corresponding numbers were 99.7% (1643/1648) and 93.8% (1545/1648), respectively. Ambiguous immunophenotyping was seen in samples that were homogenous regarding either *C4A* or *C4B*, as expected.

In samples with unambiguous results in both analyses, the copy numbers showed concordance in 95.7% for C4A (1436/1500, kappa = 0.93, p<0.001) and 97.2% for C4B CNV results (1499/1542, kappa = 0.95, p<0.001). The discrepancies were mainly cases with lower protein levels (85.9%, 55/64 for C4A and 81.4%, 35/43 for C4B, Table S4A and B). Both *C4A* and *C4B* copy numbers were divergent in 28 patients. Twenty-three of them had lower serum C4 levels than expected by genetic background. Eleven subjects had no detectable C4A protein regardless the presence of *C4A* genes (Table S4A. For C4B, the corresponding number was twelve. These may represent subjects with increased consumption, lowered production or uncharacterized mutations.

### Complement C4 Gene Frequencies in Infection Prone Patients Differ from General Population

The frequencies of *C4A*, *C4B* and *CTins* are shown in parallel with published frequencies in different populations in **Table S5**. The most common *C4* CNV was two for both *C4A* and *C4B*, detected in more than half of the cases. *CTins* was present in 6.4% (105/1618) and resulted in *C4A* deficiency (functional CNV <2) in 72% (76/105) of carriers.

**Figure 2:**
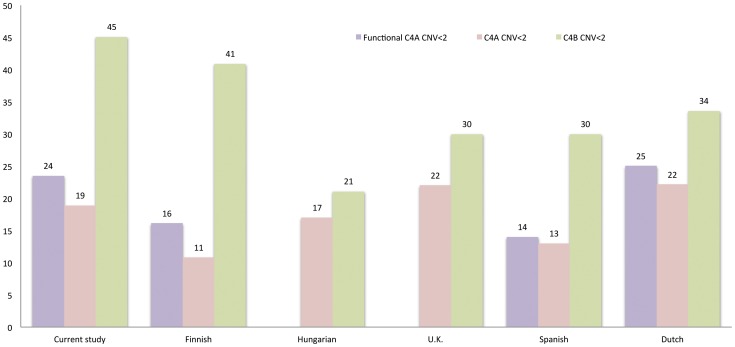
Frequencies of C4 deficiencies in different populations. The frequencies (%) of phenotypic C4A deficiency (functional C4 copy number <2, *CTins* reduced from *C4A*), *C4A* deficiency (copy number <2) and *C4B* deficiency (copy number <2) are shown. The populations are Finnish patients from the current study with unambiguous C4 qPCR results (*n* = 1618), Finnish (*n* = 149) [28], Hungarian (*n* = 118) [13], U.K. (*n* = 719) [9], Spanish (*n* = 460) [9] and Dutch (*n* = 104) [19] general population samples.

Low *C4A* CNV (<2) was recorded in 18.9% (305/1618), whereas phenotypic *C4A* deficiency (low functional CNV caused by low CNV and *CTins*) in 23.5% of study samples (381/1618) (**Figure 2**). Twenty per cents (76/381) of phenotypic *C4A* deficiency were due the presence of non-expression caused by *CTins*. Both forms of *C4A* deficiency were more frequent in patients than in a population sample of same nationality, although the low CNV seemed to cause a larger difference (OR = 1.93 95% CI = 1.13–3.29, *p* = 0.014 for low CNV and OR = 1.60, 95%CI = 1.02–2.52, *p* = 0.039 for phenotypic *C4A* deficiency, **Figure 2**). *C4B* deficiency was more frequent in patients, but the difference did not reach statistical significance.

When *C4A* and *C4B* combinations were constructed, the patients had significantly lower frequency of two copies of *C4A* without *CTins* and two copies of *C4B*, the most common combination of healthy subjects of same nationality (29% vs. 39%, respectively; p = 0.017, data not shown). *C4* CNVs of different isotypes were inversely correlated, (high *C4A* CNV was associated with low *C4B* and vice versa, data not shown) as previously stated. [19].

### Allotype (Allele) Frequencies do not Differ in Study Populations

Allotype frequencies were calculated from samples with concordant qPCR and immunophenotyping (**Table S6**). The allele distribution was essentially similar to that of a population sample of same nationality (data not shown).

## Discussion

We have developed a simple and reliable real-time qPCR assay for determining the complement *C4A*, *CTins and C4B* copy numbers. Combining the *C4A* and *CTins* results enables the assessment of different forms of *C4A* deficiencies and -related disease associations. This approach also allows the distinction between phenotypic and genotypic disease associations. The results are comparable with different methods, even with DNA isolated by different protocols [20].

SYBR® Green emits strong fluorescence on excitation when binding double stranded DNA. The amount of DNA is directly proportional to the fluorescence. Compared to TaqMan®, the SYBR® Green labelling is inexpensive, has the capability to be used with all real-time cyclers and bears a smaller risk of obtaining inconclusive results (2% vs. 5%) and is therefore an appealing choice for a diagnostic tool [14], [25]. We have used this method in disease association studies and also in assessing HLA-alleles [26]–[28].

Absolute quantification allows the direct determination of CNV. The use of many controls decreases the error rate [10], [14]. The use of concentration range allows reliable results with both comparable and slightly different concentrations without the need of internal control normalization or extensive computational procedures.

On the other hand, SYBR® Green labelling is sensitive to both DNA quality and concentration [10]. The taken precautions ensure reliable and replicable results but make the analyses more laborious and time-consuming; (i) The housekeeping gene (beta-actin) run prior to *C4* analyses controls for the DNA quality and concentration and it sets the samples in reference to *C4* controls (lower, equal or higher). The results of this analysis have to be available before starting the *C4* runs. (ii) To eliminate errors in pipetting, a run includes a duplicate of every sample and control. (iii) The fluorescence curve of every sample is individually analyzed in contrast to the controls taking into account the possible differences in concentrations. (iv) Degraded or old dilutions (over 2–3 weeks of 5 ng/µl concentration) are discarded. We are currently using sample concentrations of 10 ng/µl that are more resistant to degradation [14]. (v) Using the concentration range is a novel way to reduce erroneous CNV calling. However, the standard concentration dilutions may be prone to errors.

One run lasts for approximately 90 minutes and can include up to 44 samples. Adding 30 minutes of pipetting, a patient’s four real-time qPCR runs take 8 hours, excluding the time for making dilutions and analysing the results manually.

The optimization is critical and demanding for qPCR analyses, especially with nonspecific dyes, where the specificity relies on PCR reaction [10]. Whilst performing this, we observed that different SYBR® Green Master Mixes might vary in efficacy in different qPCR conditions (**Table 1**). This may be one of the reasons for reported qPCR difficulties [17].

The publicly available IHWIG cell lines can be used as controls, but not for *CTins*. Controls for *CTins* as well as for other runs can be obtained from the authors upon request.

*CTins* is a mutation leading to premature stop-codon and non-expression of C4 protein. If present, *CTins* causes *C4A* deficiency in more than 60% of the carriers, while it has been characterized in *C4B* in only three cases [9], [19], [21], [29], [30]. Thus, the assessment of *CTins* can be used as a screening test for *C4A* deficiencies in large materials. For individual patients, however, immunophenotyping together with genetic analyses adds valuable information. Immunophenotyping enables the detection of aberrant or non-functional allotypes (such as A6 and B45), low complement levels due to increased consumption or consistently low levels that may indicate other, rare silencing mutations. The importance of different allotypes is not known and might be a future interest.

In the patient material, *CTins* caused 20% of “functional” *C4A* deficiencies and *CTins* was present in more than six per cents. These figures correspond to earlier publications; three studies have reported the frequency of *CTins* accounting for 10–30% of C4A deficiencies in healthy population, but in one study, the rate was only 1% [16], [19], [30], [31]. *CTins* itself has been characterized in a frequency between 1–6%, but has been detected only once in Asian population [7], [9], [21], [32].

C4A is more important in the clearance of immune complexes and apoptotic cells, whereas C4B is involved in the defence against microbes [7]. Accordingly, phenotypic C4A deficiencies have been traditionally associated with susceptibility to autoimmune diseases, whereas C4B deficiencies have shown predisposition to infections with encapsulated bacteria, acute myocardial infarction and stroke [1]–[3]. Recent studies suggests that *C4A* deficiency may also be linked with increased susceptibility to infections [27], [33]. The most common *C4A-B* combination (2 *C4A* and *C4B* genes) was significantly lower in patients than in reference population, further supporting the role of abnormal C4 CNV in aberrant immune function. However, due to the lack of background information and heterogeneity of our patients, no conclusions can be drawn. We are currently conducting projects that will shed light to the potential associations between autoimmune conditions, susceptibility to infections and different forms of genetic *C4* deficiencies.

To our knowledge, the differences in disease associations between C4 deficiency due to low CNV or phenotypic deficiencies due to low CNV and *CTins* have not been assessed before. In a study in hepatitis virus B non-responders, C4A deficiency was more common in non-responders, whereas *CTins* in responders [34]. The strong linkage between *CTins* and HLA-DRB1*13 could explain the seemingly contradictory findings [8], [9], [34]. *CTins* was twice but non-significantly more common in patients with meningococcal disease, compared with controls [19]. In SLE, the frequency of *CTins* was similar in cases and controls [35].

### Conclusion

We describe a simple approach for determination of *C4* gene copy numbers and deficiencies due to *CTins*. This method exhibits comparability with other methods and has the advantage of high throughput and absolute quantification. The parallel use of *CTins* and *C4* CNV analyses increases the detection rate of *C4A* deficiency, and could be used as a screening tool. In individual patient cases, qPCR combined with immunophenotyping provides information of the personalized C4 status in relation to gene deletion, mutation, presence of non-functional allotypes or increased C4 consumption and is useful in the assessment of immune deficiencies.

## Supporting Information

Table S1 **A–C Detailed qPCR results of samples in method validation, specified by runs and dilutions.**
(DOC)

Table S2 **Published and observed results of complement** ***C4A*** **and** ***C4B*** **copy numbers.**
(DOC)

Table S3 **Prevention of false interpretation of CNVs by the use of concentration range.**
(DOC)

Table S4 **A and B Comparison of** ***C4*** **copy number variation (CNV) results between qPCR and immunophenotyping.**
(DOC)

Table S5 **Complement C4 genotype frequencies (%) in different Caucasian populations.**
(DOC)

Table S6 **A and B C4 allotype frequencies detailed by copy numbers.**
(DOC)

## References

[1] Samano ES, Ribeiro Lde M, Gorescu RG, Rocha KC, Grumach AS (2004). Involvement of C4 allotypes in the pathogenesis of human diseases.. Rev Hosp Clin Fac Med Sao Paulo.

[2] Szilagyi A, Fust G (2008). Diseases associated with the low copy number of the C4B gene encoding C4, the fourth component of complement.. Cytogenet Genome Res.

[3] Mougey R (2010). A review of the Chido/Rodgers blood group.. Immunohematology.

[4] Zafar GI, Grimm EA, Wei W, Johnson MM, Ellerhorst JA (2009). Genetic deficiency of complement isoforms C4A or C4B predicts improved survival of metastatic renal cell carcinoma.. J Urol 181(3): 1028–34; discussion 1034.

[5] Yu CY, Belt KT, Giles CM, Campbell RD, Porter RR (1986). Structural basis of the polymorphism of human complement components C4A and C4B: Gene size, reactivity and antigenicity.. EMBO J.

[6] Braun L, Schneider PM, Giles CM, Bertrams J, Rittner C (1990). Null alleles of human complement C4. evidence for pseudogenes at the C4A locus and for gene conversion at the C4B locus.. J Exp Med.

[7] Blanchong CA, Chung EK, Rupert KL, Yang Y, Yang Z (2001). Genetic, structural and functional diversities of human complement components C4A and C4B and their mouse homologues, slp and C4.. Int Immunopharmacol.

[8] Barba G, Rittner C, Schneider PM (1993). Genetic basis of human complement C4A deficiency. detection of a point mutation leading to nonexpression.. J Clin Invest.

[9] Boteva L, IMAGEN, Wu YL, Cortes-Hernandez J, Martin J (2011). Determination of the loss of function complement C4 exon 29 CT insertion using a novel paralog-specific assay in healthy UK and spanish populations.. PLoS One.

[10] D’haene B, Vandesompele J, Hellemans J (2010). Accurate and objective copy number profiling using real-time quantitative PCR.. Methods.

[11] Gouas L, Goumy C, Veronese L, Tchirkov A, Vago P (2008). Gene dosage methods as diagnostic tools for the identification of chromosome abnormalities.. Pathol Biol (Paris).

[12] Lee JH, Jeon JT (2008). Methods to detect and analyze copy number variations at the genome-wide and locus-specific levels.. Cytogenet Genome Res.

[13] Szilagyi A, Blasko B, Szilassy D, Fust G, Sasvari-Szekely M (2006). Real-time PCR quantification of human complement C4A and C4B genes.. BMC Genet.

[14] Wu YL, Savelli SL, Yang Y, Zhou B, Rovin BH (2007). Sensitive and specific real-time polymerase chain reaction assays to accurately determine copy number variations (CNVs) of human complement C4A, C4B, C4-long, C4-short, and RCCX modules: Elucidation of C4 CNVs in 50 consanguineous subjects with defined HLA genotypes.. J Immunol.

[15] Wu YL, Hauptmann G, Viguier M, Yu CY (2009). Molecular basis of complete complement C4 deficiency in two north-african families with systemic lupus erythematosus.. Genes Immun.

[16] Blanchong CA, Zhou B, Rupert KL, Chung EK, Jones KN (2000). Deficiencies of human complement component C4A and C4B and heterozygosity in length variants of RP-C4-CYP21-TNX (RCCX) modules in caucasians. the load of RCCX genetic diversity on major histocompatibility complex-associated disease.. J Exp Med.

[17] Chung EK, Yang Y, Rupert KL, Jones KN, Rennebohm RM (2002). Determining the one, two, three, or four long and short loci of human complement C4 in a major histocompatibility complex haplotype encoding C4A or C4B proteins.. Am J Hum Genet.

[18] Wu YL, Yang Y, Chung EK, Zhou B, Kitzmiller KJ (2008). Phenotypes, genotypes and disease susceptibility associated with gene copy number variations: Complement C4 CNVs in european american healthy subjects and those with systemic lupus erythematosus.. Cytogenet Genome Res.

[19] Wouters D, van Schouwenburg P, van der Horst A, de Boer M, Schooneman D (2009). High-throughput analysis of the C4 polymorphism by a combination of MLPA and isotype-specific ELISA’s.. Mol Immunol.

[20] Fernando MM, Boteva L, Morris DL, Zhou B, Wu YL (2010). Assessment of complement C4 gene copy number using the paralog ratio test.. Hum Mutat.

[21] Ittiprasert W, Kantachuvesiri S, Pavasuthipaisit K, Verasertniyom O, Chaomthum L (2005). Complete deficiencies of complement C4A and C4B including 2-bp insertion in codon 1213 are genetic risk factors of systemic lupus erythematosus in thai populations.. J Autoimmun.

[22] Yu CY (1991). The complete exon-intron structure of a human complement component C4A gene. DNA sequences, polymorphism, and linkage to the 21-hydroxylase gene.. J Immunol.

[23] Sim E, Cross SJ (1986). Phenotyping of human complement component C4, a class-III HLA antigen.. Biochem J.

[24] Zhang WJ, Kay PH, Cobain TJ, Dawkins RL (1988). C4 allotyping on plasma or serum: Application to routine laboratories.. Hum Immunol.

[25] Scheurer ME, Dillon LM, Chen Z, Follen M, Adler-Storthz K (2007). Absolute quantitative real-time polymerase chain reaction for the measurement of human papillomavirus E7 mRNA in cervical cytobrush specimens.. Infect Agent Cancer.

[26] Wennerström A, Pietinalho A, Vauhkonen H, Lahtela L, Palikhe A, Hedman J (2012). HLA-DRB1 allele frequencies and C4 copy number variation in Finnish sarcoidosis patients and associations with disease prognosis.. Hum Immunol.

[27] Kainulainen L, Peltola V, Seppänen M, Viander M, He Q, Lokki ML, Ruuskanen O (2012). C4A deficiency in children and adolescents with recurrent respiratory infections.. Hum Immunol.

[28] Paakkanen R, Lokki ML, Seppanen M, Tierala I, Nieminen MS (2012). Proinflammatory HLA-DRB1*01-haplotype predisposes to ST-elevation myocardial infarction.. Atherosclerosis.

[29] Lokki ML, Circolo A, Ahokas P, Rupert KL, Yu CY (1999). Deficiency of human complement protein C4 due to identical frameshift mutations in the C4A and C4B genes.. J Immunol.

[30] Seppanen M, Lokki ML, Notkola IL, Mattila K, Valtonen V (2007). Complement and c4 null alleles in severe chronic adult periodontitis.. Scand J Immunol.

[31] Sullivan KE, Kim NA, Goldman D, Petri MA (1999). C4A deficiency due to a 2 bp insertion is increased patients with systemic lupus erythematosus.. J Rheumatol.

[32] Puah SM, Lian LH, Chew CH, Chua KH, Tan SY (2007). A study of association of the complement C4 mutations with systemic lupus erythematosus in the malaysian population.. Lupus.

[33] Senbagavalli P, Kumar N, Kaur G, Mehra NK, Geetha ST (2011). Major histocompatibility complex class III (C2, C4, factor B) and C3 gene variants in patients with pulmonary tuberculosis.. Hum Immunol.

[34] Hohler T, Stradmann-Bellinghausen B, Starke R, Sanger R, Victor A (2002). C4A deficiency and nonresponse to hepatitis B vaccination.. J Hepatol.

[35] Boteva L, Morris DL, Cortés-Hernández J, Martin J, Vyse T (2012). Genetically determined partial complement C4 deficiency states are not independent risk factors for SLE in UK and Spanish populations.. Am J Hum Gen.

