# Correction: Copy Number Analysis of Complement *C4A*, *C4B* and *C4A* Silencing Mutation by Real-Time Quantitative Polymerase Chain Reaction

**DOI:** 10.1371/annotation/d19ba035-dbf0-4e58-932a-52efaa8137f3

**Published:** 2012-09-28

**Authors:** Riitta Paakkanen, Hanna Vauhkonen, Katja T. Eronen, Asko Järvinen, Mikko Seppänen, Marja-Liisa Lokki

There was an error in the Competing Interests statement. The correct Competing interests are:

MLL is working as a consultant at a company owned by the University of Helsinki providing complement C4 analysis for diagnostic samples. This does not alter the authors' adherence to all the PLoS ONE policies on sharing data and materials. The other authors have no competing interests to declare. 

