# Supplementary material for: Copy Number Analysis of Complement C4A, C4B and C4A Silencing Mutation by Real-Time Quantitative Polymerase Chain Reaction
Source: PLoS One. 2012 Jun 21;7(6):e38813. doi: 10.1371/journal.pone.0038813 (PMC3380926; doi:10.1371/journal.pone.0038813)
Supplement: Table S1 — A–C Detailed qPCR results of samples in method validation, specified by runs and dilutions. (DOC) [file pone.0038813.s001.doc]

| **Supplementary Table S1A.** Detailed *C4A* qPCR results of samples in method validation, specified by runs and dilutions. | | | | | | | | | | | | | | |
| --- | --- | --- | --- | --- | --- | --- | --- | --- | --- | --- | --- | --- | --- | --- |
|  |  |  | **Ct values by run (mean)** | | | | |  | **Ct values by dilution** | | | | | |
|  |  |  | **C4A run*** | | | | |  | **A** | | | **B** | | |
|  |  | **C4A CNV** | **1** | **2** | **3** | **4** | **5** |  | **Mean** | **SD** | **Range** | **Mean** | **SD** | **Range** |
| ID | TX-1586 | 1 | 21.2 | 21.2 | 21.3 | 21.5 | 21.6 |  | 21.3 | 0.13 | 0.4 | 21.6 | 0.19 | 0.6 |
|  | TX-2144 | 2 | 20.3 | 20.2 | 20.5 | 20.2 | 20.3 |  | 20.4 | 0.2 | 0.6 | 20.2 | 0.08 | 0.3 |
|  | TX-2147 | 3 | 19.2 | 19.1 | 19.2 | 19.3 | 19.3 |  | 19.2 | 0.08 | 0.3 | 19.3 | 0.19 | 0.7 |
|  | TX-2158 | 1 | 21.3 | 21.2 | 21.3 | 21.3 | 21.4 |  | 21.3 | 0.07 | 0.3 | 21.4 | 0.17 | 0.6 |
|  | TX-2170 | 2 | 20.3 | 20.2 | 20.5 | 20.5 | 20.8 |  | 20.3 | 0.14 | 0.5 | 20.6 | 0.3 | 1.1 |
|  | TX-2209 | 0 | . | . | . | . | . |  | . | . | . | . | . | . |
|  | TX-2284 | 3 | 19.8 | 19.9 | 19.9 | 19.9 | 20.1 |  | 19.8 | 0.08 | 0.3 | 20 | 0.22 | 0.8 |
| R^2^ value | |  | 0.92 | 0.91 | 0.94 | 0.88 | 0.81 |  |  |  |  |  |  |  |
| Efficiency | |  | 0.89 | 0.91 | 0.92 | 0.82 | 0.86 |  |  |  |  |  |  |  |
| Replicates (n) | |  | 2 | 6 | 6 | 6 | 6 |  | 14 | | | 12 | | |
| Dilution* | |  | A | A | A | B | B |  |  |  |  |  |  |  |

Abbreviations:

C4A (complement component C4A),

CNV (copy number variation),

qPCR (real-time quantitative PCR),

R^2^ (the correlation between given data and obtained data).

Efficiency (the percentage of duplicated DNA per cycle).

Ct value (cycle threshold value, the cut-off point where samples and controls are compared for differences in concentrations).

* Method validation was performed from two independent dilutions (A and B) in independent replicates in five runs.

| **Supplementary Table S1B.** Detailed *C4B* qPCR results of samples in method validation, specified by runs and dilutions | | | | | | | | | | | | | | | | |
| --- | --- | --- | --- | --- | --- | --- | --- | --- | --- | --- | --- | --- | --- | --- | --- | --- |
|  |  |  | **Mean Ct by qPCR run** | | | | | |  | **Ct values by dilution** | | | | | | |
|  |  | **C4B CNV** | C4B run* | | | | | |  | **A** | | | **B** | | | |
|  |  |  | **1** | **2** | **3** | **4** | **5** |  | | **Mean** | **SD** | **Range** | | **Mean** | **SD** | **Range** |
| ID | TX-1586 | 3 | 19.1 | 19.2 | 19.4 | 19.6 | 20.1 |  | | 19.3 | 0.17 | 0.6 | | 19.9 | 0.28 | 0.8 |
|  | TX-2144 | 0 | . | . | . | . | . |  | | . | . | . | |  | . |  |
|  | TX-2147 | 0 | . | . | . | . | . |  | | . | . | . | |  | . |  |
|  | TX-2158 | 2 | 20 | 20 | 20 | 20.1 | 20.6 |  | | 20 | 0.1 | 0.3 | | 20.3 | 0.3 | 1 |
|  | TX-2170 | 3 | 19.4 | 19.4 | 19.6 | 19.7 | 20.2 |  | | 19.5 | 0.22 | 1 | | 20 | 0.36 | 1.1 |
|  | TX-2209 | 2 | 20.1 | 20.2 | 20.2 | 20.3 | 20.8 |  | | 20.2 | 0.07 | 0.3 | | 20.6 | 0.26 | 0.8 |
|  | TX-2284 | 1 | 20.8 | 21 | 20.9 | 21 | 21.5 |  | | 20.9 | 0.14 | 0.5 | | 21.2 | 0.29 | 1 |
| R^2^ |  |  | 0.96 | 0.94 | 0.88 | 0.93 | 0.8 |  | |  |  |  | |  |  |  |
| Efficiency | |  | 1 | 0.91 | 1.21 | 1.25 | 1.36 |  | |  |  |  | |  |  |  |
| Replicates(n) | |  | 2 | 6 | 6 | 6 | 6 |  | | 14 | | | | 12 | | |
| Dilution* | |  | A | A | A | B | B |  | |  |  |  | |  |  |  |

Abbreviations:

C4B (complement component C4B),

CNV (copy number variation),

qPCR (real-time quantitative PCR),

R^2^ (the correlation between given data and obtained data).

Efficiency (the percentage of duplicated DNA per cycle).

Ct value (cycle threshold value, the cut-off point where samples and controls are compared for differences in concentrations).

* Method validation was performed from two independent dilutions (A and B) in independent replicates in five runs.

| **Supplementary Table S1C.** Detailed *CTins* qPCR results of samples in method validation, specified by runs and dilutions | | | | | | | | | | | | | |
| --- | --- | --- | --- | --- | --- | --- | --- | --- | --- | --- | --- | --- | --- |
|  |  |  | Ct mean by run | | | | | |  | Ct-values by dilution | | | |
|  |  | CTins CNV | *CTins* run | |  |  |  |  |  | A | | B | |
|  |  |  | 1 | 2 | 3 | 4 | 5 | 6 |  | Mean | SD | Mean | SD |
| ID | TX-1586 | 0 | . | . | . | . | . | . |  | . | . | . | . |
|  | TX-2144 | 1 | 24.4 | 24.3 | 24.3 | 24.8 | 24.4 | 24.5 |  | 24.5 | 0.43 | 24.5 | 0.45 |
|  | TX-2147 | 0 | . | . | . | . | . | . |  | . | . | . | . |
|  | TX-2158 | 1 | 24.2 | 24.7 | 24.7 | 24.3 | 24.5 | 24.3 |  | 24.5 | 0.41 | 24.4 | 0.39 |
|  | TX-2170 | 0 | . | . | . | . | . | . |  | . | . | . | . |
|  | TX-2209 | 0 | . | . | . | . | . | . |  | . | . | . | . |
|  | TX-2284 | 0 | . | . | . | . | . | . |  | . | . | . | . |
| Dilution |  |  | A | A | A | A | B | B |  |  |  |  |  |
| Replicates (n) | |  | 6 | 6 | 6 | 6 | 6 | 6 |  | 24 | | 12 | |

Abbreviations:

CTins (silencing mutation of complement component C4A),

CNV (copy number variation),

qPCR (real-time quantitative PCR),

R^2^ (the correlation between given data and obtained data).

Efficiency (the percentage of duplicated DNA per cycle).

Ct value (cycle threshold value, the cut-off point where samples and controls are compared for differences in concentrations).
