# Supplementary material for: Copy Number Analysis of Complement C4A, C4B and C4A Silencing Mutation by Real-Time Quantitative Polymerase Chain Reaction
Source: PLoS One. 2012 Jun 21;7(6):e38813. doi: 10.1371/journal.pone.0038813 (PMC3380926; doi:10.1371/journal.pone.0038813)
Supplement: Table S2 — Published and observed results of complement C4A and C4B copy numbers. (DOC) [file pone.0038813.s002.doc]

| **Supplementary Table S2.** Published and observed results of complement *C4A* and *C4B* copy numbers. | | | | | | |
| --- | --- | --- | --- | --- | --- | --- |
| **IHWG cell line** [14] | **Reference results** | |  | **Observed copy number (Ct range)** | | |
|  | C4A | C4B |  | C4A | C4B | CTins^†^ |
| HOM2 | 3 | 2 |  | 3 (20.18-20.24) | 2 (20.72-20.96) | 0 |
| WT8 | 1 | 2 |  | 1 (22.03-22.29) | 2 (21.02-21.15) | 0 |
| COX | 0 | 2 |  | 0 | 2 (20.93-20.94) | 0 |
| JVM | 2 | 0 |  | 2 (21.01-21.03) | 0 | 0 |
| 31227ABO | 4 | 2 |  | 4 (19.83-19.88) | 2 (20.88-20.97) | 0 |
| MANIKA | 2 | 3 |  | 2 (20.83-21.34) | 3 (19.74-20.01) | 0 |

Abbreviations:

IHWG (International Histocompatibility Working Group),

*CTins* (Silencing mutation of *C4A*).

*^†^ CTins*, a silencing mutation of *C4A* was not previous studied.
