# Supplementary material for: Copy Number Analysis of Complement C4A, C4B and C4A Silencing Mutation by Real-Time Quantitative Polymerase Chain Reaction
Source: PLoS One. 2012 Jun 21;7(6):e38813. doi: 10.1371/journal.pone.0038813 (PMC3380926; doi:10.1371/journal.pone.0038813)
Supplement: Table S3 — Prevention of false interpretation of CNVs by the use of concentration range. (DOC) [file pone.0038813.s003.doc]

| **Supplementary Table S3.** Prevention of false interpretation of CNVs by the use of concentration range. | | | | | |
| --- | --- | --- | --- | --- | --- |
| **Real**  **CNV** | **False CNV interpretation** | **X-fold change in the amount of DNA needed for false interpretation** | **Concentration for the false interpretation (ng/ul)*** | **Action by beta-actin run**** |  |
| 1 | 2 | 2.0 | 20.0 | discard |  |
| 2 | 1 | 0.5 | 5.0 | discard |  |
|  | 3 | 1.5 | 15.0 | discard |  |
|  | 4 | 2.0 | 20.0 | discard |  |
| 3 | 1 | 0.3 | 3.0 | discard |  |
|  | 2 | 0.6 | 6.0 | discard |  |
|  | 4 | 1.3 | 13.0 | re-dilute |  |
| 4 | 1 | 0.25 | 2.5 | discard |  |
|  | 2 | 0.5 | 5.0 | discard |  |
|  | 3 | 0.75 | 7.5 | discard |  |
|  | 5 | 1.25 | 12.5 | re-dilute |  |
| Abbreviation: | |  |  |  |  |
| CNV (copy number variation) | |  |  |  |  |
| * Result of X-fold change in the sample concentration of 10 ng/ul. | | | | | |
| ** Beta-actin inclusion range is 8-14 ng/ul. Samples within this range, but differing from the control's concentration are used, but rounded up or down to compensate for the difference. | | | | | |
