# Supplementary material for: Copy Number Analysis of Complement C4A, C4B and C4A Silencing Mutation by Real-Time Quantitative Polymerase Chain Reaction
Source: PLoS One. 2012 Jun 21;7(6):e38813. doi: 10.1371/journal.pone.0038813 (PMC3380926; doi:10.1371/journal.pone.0038813)
Supplement: Table S4 — A and B Comparison of C4 copy number variation (CNV) results between qPCR and immunophenotyping. (DOC) [file pone.0038813.s004.doc]

| **Supplementary Table S4A.** Comparison of C4A copy number variation (CNV) results between qPCR and immunophenotyping. | | | | | | |
| --- | --- | --- | --- | --- | --- | --- |
| C4A CNV by qPCR* | C4A CNV by phenotyping | | | | | Total (n) |
|  | 0 | 1 | 2 | 3 | 4 |  |
| 0 | **39** | 0 | 0 | 0 | 0 | 39 |
| 1 | *5* | **323** | 4 | 0 | 0 | 332 |
| 2 | *4* | *8* | **690** | 5 | 0 | 707 |
| 3 | *2* | 0 | *32* | **378** | 0 | 412 |
| 4 | 0 | 0 | *1* | *3* | **6** | 10 |
| Total (n) | 50 | 331 | 727 | 386 | 6 | 1500 |
| Abbreviations: | |  |  |  |  |  |
| qPCR (real-time quantitative PCR), | | |  |  |  |  |
| C4A (complement component C4A), | | |  |  |  |  |
| CNV (copy number variation). | | |  |  |  |  |
| ^*^ *C4A* silencing mutation has been reduced from C4A CNVs. | | | | |  |  |
| The number of lower CNV by genotyping (in italic) is 55. | | | | |  |  |

| **Supplementary Table S4B.** Comparison of *C4B* copy number variation (CNV) results between qPCR and immunophenotyping. | | | | | |
| --- | --- | --- | --- | --- | --- |
| C4B CNV by qPCR | C4B CNV by phenotyping | | | | Total (n) |
|  | 0 | 1 | 2 | 3 |  |
| 0 | **116** | 0 | 0 | 0 | 116 |
| 1 | *1* | **602** | 7 | 0 | 610 |
| 2 | *11* | *15* | **773** | 1 | 800 |
| 3 | 0 | 0 | *7* | **8** | 15 |
| Total (n) | 128 | 617 | 787 | 9 | 1541***** |
| Abbreviations: | |  |  |  |  |
| qPCR (real-time quantitative PCR), | | |  |  |  |
| C4B (complement component C4B), | | |  |  |  |
| CNV (copy number variation). | | |  |  |  |
| *One case with C4B CNV=4 with qPCR and CNV=3 in immunophenotyping was excluded. | | | | | |
| The number of lower CNV by genotyping (samples in italic plus one excluded case*) is 35. | | | | | |
