# Supplementary material for: Copy Number Analysis of Complement C4A, C4B and C4A Silencing Mutation by Real-Time Quantitative Polymerase Chain Reaction
Source: PLoS One. 2012 Jun 21;7(6):e38813. doi: 10.1371/journal.pone.0038813 (PMC3380926; doi:10.1371/journal.pone.0038813)
Supplement: Table S5 — Complement C4 genotype frequencies (%) in different Caucasian populations. (DOC) [file pone.0038813.s005.doc]

| Supplementary Table S5. Complement C4 genotype frequencies (%) in different Caucasian populations. | | | | | | | |
| --- | --- | --- | --- | --- | --- | --- | --- |
|  |  | Current study n=1618[[1]](#footnote-2) | Finnish [28] n=149 | Hungarian [13] n=118 | U.K. [9] n=719[[2]](#footnote-3) | Spanish [9] n=449^†^ | Dutch [19] n=104 |
| *C4A* copy number | 0 | 1.3 | 0.7 | 1.7 | 1 | 1 | 5.77 |
|  | 1 | 17.6 | 10.1 | 15.3 | 21 | 12 | 16.35 |
|  | 2 | 51.1 | 57.7 | 55.1 | 52 | 58 | 50.96 |
|  | 3 | 29 | 28.9 | 25.4 | 20 | 5 | 23.08 |
|  | 4 | 1.1 | 2.7 | 2.5 | 6 | 4 | 3.85 |
|  | >5 | 0 | 0 | 0 | 0 | 1 | 0 |
| TC insertion (n) | 1 | 6.2 | 6.7 | . | 3.8 | 1.8 | 4.8 |
|  | 2 | 0.2 | 0 | . | 0.3 | 0 | 0 |
|  | 3 | 0 | 0 |  | 0.1 | 0 | 0 |
| *C4B* copy number | 0 | 7 | 10.1 | 0.8 | 4 | 3 | 4.8 |
|  | 1 | 38.1 | 30.9 | 20.3 | 26 | 27 | 28.8 |
|  | 2 | 53.5 | 58.4 | 63.6 | 57 | 51 | 59.6 |
|  | 3 | 1.4 | 0.7 | 15.3 | 13 | 14 | 6.7 |
|  | 4 | 0.1 | 0 | 0 | 0 | 5 | 0 |
|  |  |  |  |  |  |  |  |
|  |  |  |  |  |  |  |  |
|  |  |  |  |  |  |  |  |
| Method |  | SYBR® Green qPCR | Scorpion and SYBR® Green qPCR | TaqMan® qPCR | Paralog test / REDVR for *CTins* | Paralog test REDVR for *CTins* | MPLA |

1. The reference numbers refer to the references in the manuscript.

   Abbreviations:

   qPCR (real-time quantitative polymerase chain reaction),

   Deficiency (CNV <2),

   Functional C4A deficiency (functional CNV<2, where the CTins-silenced copies of C4A are considered as absent),

   TaqMan®, Scorpion (specific qPCR dyes),

   REDVR (Restriction enzyme digest variant ratio analysis),

   MPLA (Multiplex Ligation-dependent Probe Amplification).

   ? Unambiguous results in all real-time quantitative PCR (qPCR) analyses.  [↑](#footnote-ref-2)
2. Values estimated from graphical presentation. [↑](#footnote-ref-3)
