# Supplementary material for: Copy Number Analysis of Complement C4A, C4B and C4A Silencing Mutation by Real-Time Quantitative Polymerase Chain Reaction
Source: PLoS One. 2012 Jun 21;7(6):e38813. doi: 10.1371/journal.pone.0038813 (PMC3380926; doi:10.1371/journal.pone.0038813)
Supplement: Table S6 — A and B C4 allotype frequencies detailed by copy numbers. (DOC) [file pone.0038813.s006.doc]

| **Supplementary Table S6A.** C4A allotype frequencies detailed by copy numbers | | | |
| --- | --- | --- | --- |
| **C4A CNV** | **Allotype** | **n** | **Frequency^*^** |
| 1 |  | 322 | 1 |
|  | 2 | 11 | 0.03 |
|  | 3 | 259 | 0.80 |
|  | 4 | 43 | 0.13 |
|  | 6 | 9 | 0.03 |
|  |  |  |  |
| 2 |  | 690 | 1.00 |
|  | 3,1 | 2 | 0.00 |
|  | 3,2 | 70 | 0.10 |
|  | 3,3 | 452 | 0.66 |
|  | 3,6 |  | 0.00 |
|  | 4,2 | 8 | 0.01 |
|  | 4,3 | 94 | 0.14 |
|  | 4,4 | 34 | 0.05 |
|  | 6,3 | 28 | 0.04 |
|  | 6,4 | 2 | 0.00 |
|  |  |  |  |
| 3 |  | 379 | 1.00 |
|  | 3,2,2 | 15 | 0.04 |
|  | 3,3,2 | 179 | 0.47 |
|  | 3,3,3 | 142 | 0.37 |
|  | 4,3,1 | 1 | 0.00 |
|  | 4,3,2 | 30 | 0.08 |
|  | 4,3,3 | 4 | 0.01 |
|  | 5.5,3,2 | 1 | 0.00 |
|  | 6,3,2 | 4 | 0.01 |
|  | 6,3,3 | 2 | 0.01 |
|  |  |  |  |
| 4 |  | 6 | 1.00 |
|  | 3,3,2,2 | 4 | 0.67 |
|  | 3,3,3,2 | 2 | 0.33 |

CNV (copy number variation)

^*^1397 patients with copy number>0 and concordant quantitative PCR and immunophenotyping results are included.

| **Supplementary Table S6B.** C4B allotype frequencies detailed by copy number | | | |
| --- | --- | --- | --- |
| **C4B CNV** | **Allotype** | **n** | **Frequency^*^** |
| 1 |  | 602 | 1 |
|  | 1 | 455 | 0.76 |
|  | 2 | 118 | 0.20 |
|  | 3 | 24 | 0.04 |
|  | 5 | 5 | 0.01 |
|  |  |  |  |
| 2 |  | 773 | 1.00 |
|  | 1,1 | 386 | 0.50 |
|  | 1,2 | 259 | 0.34 |
|  | 1,3 | 72 | 0.09 |
|  | 1,5 | 5 | 0.01 |
|  | 1.9,3 | 1 | 0.00 |
|  | 2,2 | 40 | 0.05 |
|  | 2,3 | 5 | 0.01 |
|  | 2,5 | 2 | 0.00 |
|  | 3,3 | 2 | 0.00 |
|  | 3,5 | 1 | 0.00 |
|  |  |  |  |
| 3 |  | 8 | 1.00 |
|  | 1,1,1 | 3 | 0.38 |
|  | 1,1,2 | 1 | 0.13 |
|  | 1,2,2 | 1 | 0.13 |
|  | 1,2,5 | 1 | 0.13 |
|  | 1,3,5 | 1 | 0.13 |
|  | 2,2,1 | 1 | 0.13 |

CNV (copy number variation)

^*^1383 patients with copy number>0 and concordant quantitative PCR and immunophenotyping results are included.
